# Off-label use of canakinumab in pediatric rheumatology and rare diseases

**DOI:** 10.3389/fmed.2022.998281

**Published:** 2022-10-18

**Authors:** Emanuela Del Giudice, Jurgen Sota, Francesca Orlando, Ludovica Picciano, Rolando Cimaz, Luca Cantarini, Angela Mauro

**Affiliations:** ^1^Pediatric and Neonatology Unit, Maternal and Child Department, Sapienza University of Rome, Polo Pontino, Latina, Italy; ^2^Department of Medical Sciences, Surgery and Neurosciences, Research Center of Systemic Autoinflammatory Diseases and Behçet's Disease Clinic, University of Siena, Siena, Italy; ^3^Pediatric Rheumatology Unit, Department of General and Emergency Pediatrics, Santobono-Pausilipon Children's Hospital, Naples, Italy; ^4^Pediatric Emergency and Short Stay Unit, Santobono-Pausilipon Children's Hospital, Naples, Italy; ^5^Department of Clinical Sciences and Community Health, University of Milan, Milan, Italy; ^6^Pediatric Rheumatology Unit, Department of Childhood and Developmental Medicine, Fatebenefratelli-Sacco Hospital, Milan, Italy

**Keywords:** canakinumab, off-label use, hereditary autoinflammatory disease, multifactorial autoinflammatory disorder, children

## Abstract

Since the first success of interleukin-1 blockade in cryopyrin-associated periodic syndrome, the use of interleukin-1 inhibitors has expanded to other disorders, including off-label indications. In particular, canakinumab has been employed in an off-label fashion in several diseases such as rare monogenic autoinflammatory diseases and multifactorial autoinflammatory diseases, disclosing an excellent efficacy and good safety profile in pediatric patients unresponsive to standards of care. In addition, hyperferritinemic syndromes and complex disorders, as well as Kawasaki disease, uveitis, and other pediatric rare disorders, represent additional areas where canakinumab efficacy is worth exploring. Altogether, the results summarized below are of paramount importance in pediatric patients where a considerable proportion of treatments are prescribed off-label. This review focuses on the off-label use of canakinumab in pediatric patients affected by systemic immune-mediated diseases.

## Introduction

The development of new drugs and their use in an off-label fashion define a substantial attempt to relieve the socioeconomic and psychological burden of rare diseases, which taken together affect a considerable proportion of the overall population. The treatment approach in rare diseases is mostly covered by the off-label practice. This is particularly true for pediatric patients due to ethical and methodological obstacles that often arise when trying to prepare and conduct randomized controlled trials. Indeed, in pediatrics, off-label treatment represents roughly half of the total number of treatment prescriptions (1), reaching more than 90% in certain areas such as neonate intensive care units (2).

Nationwide multicenter observational studies and surveys in Europe have disclosed interesting results regarding the use of interleukin (IL)-1 inhibitors. In detail, Vitale et al. (3) retrospectively collected demographic, clinical, and therapeutic data from both adult and pediatric patients treated with IL-1 inhibitors from January 2008 to July 2016 in 23 Italian hospitals. In their experience, most of the treatment courses were administered on an off-label regimen. Canakinumab (CAN) was administered in 105 (19.9%) patients, with 56% of them receiving the drug off-label. Conditions requiring off-label use included Behçet's syndrome (BS), epidermolysis bullosa, idiopathic uveitis, periodic fever, aphthous stomatitis, pharyngitis cervical adenitis (PFAPA) syndrome, vasculitic urticaria, and undifferentiated systemic autoinflammatory diseases (AIDs). Rossi-Semerano et al. analyzed the efficacy and safety of anti-IL-1 agents and collected descriptive data in a nationwide survey in France on both adult and pediatric patients receiving anti-IL-1 agents for off-label indications. CAN was used in 25 patients (18 children), and the off-label administrations included vasculitis, Schnitzler syndrome, Erdheim–Chester, and Blau syndromes (4). Also, Sota et al. described the use of CAN in real-life settings in adults and pediatric patients, including 111 children, enrolled in 23 Italian tertiary referral centers retrospectively reviewed, showing an excellent safety profile (5).

Interestingly, CAN represented the most common biotechnological drug used off-label in a Turkish study conducted at a national level on 4,992 children with rare diseases (6).

The aim of this review is to describe the currently available literature for the off-label use of CAN in pediatric patients suffering from immune-mediated disorders.

## Methods

### Search methods

This narrative review was conducted in accordance with the IMRAD (Introduction, Methods, Results, and Discussion) approach (7). A comprehensive search was performed in the bibliographic database PubMed from 2011 to 2022.

A combination of MeSH terms and keywords was employed to retrieve relevant articles serving the purposes of this review. In particular, the following words were employed: (“canakinumab”[Supplementary Concept] OR “canakinumab”[All Fields]) AND (“interleukin-1 blockade” OR “IL-1 blockade” OR “anti-interleukin-1 agents” OR “anti-IL-1 agents”) AND (“Child”[MeSH Terms] OR “Children” OR “Childhood” OR “Juvenile” OR “Pediatric” OR “Pediatric” OR “Pediatric age” OR “Pediatric age”). Duplicate articles were excluded.

**Figure 1:**
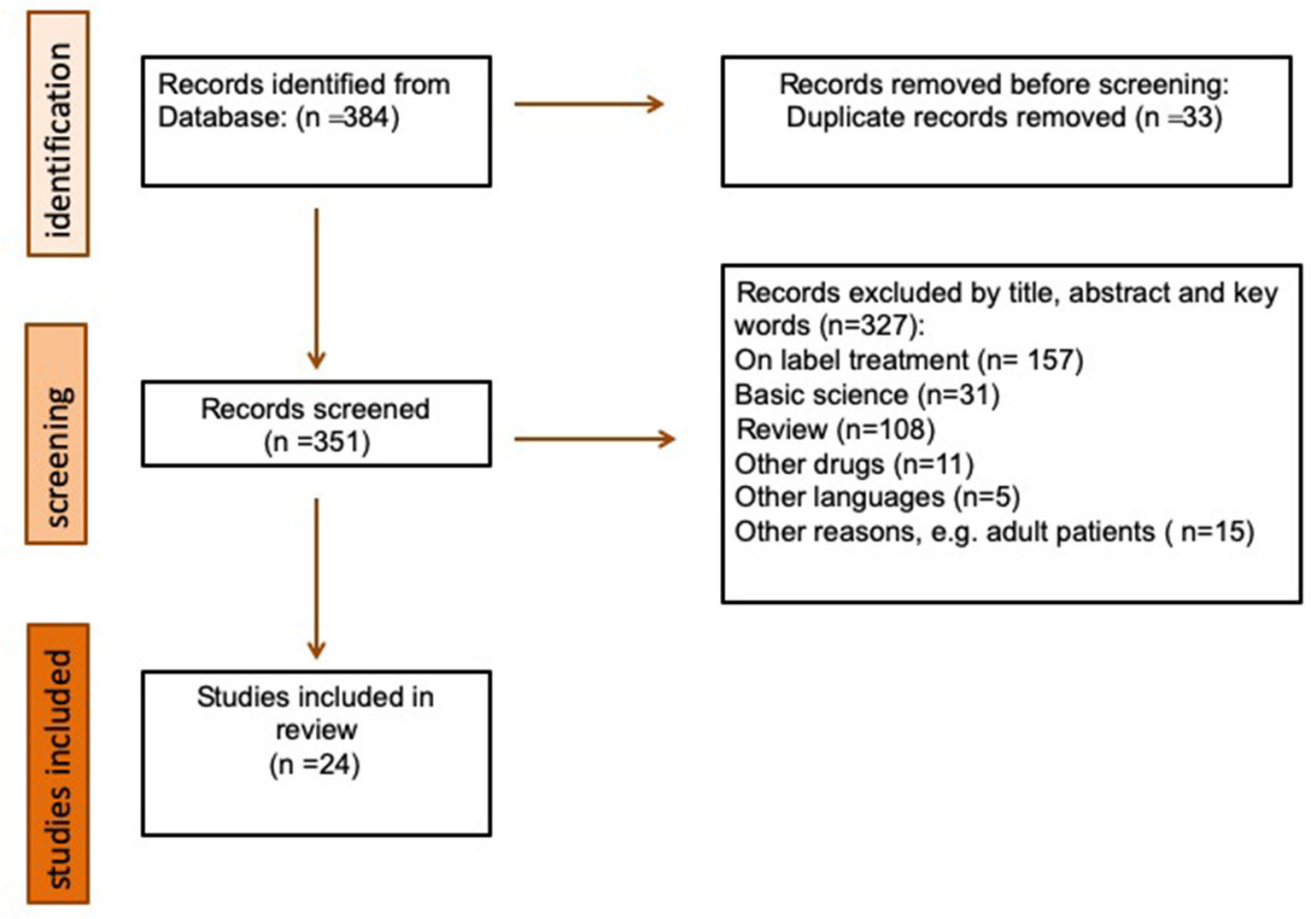
Flowchart of the literature search and study selection process.

Figure 1 shows the flow of information through the different phases of our search. It depicts the number of records identified, included, and excluded, and the reasons for exclusion.

### Selection criteria

The inclusion criterion was the off-label designation of the treatment, defined as the prescription of CAN in conditions other than that for which it has been officially approved, as listed in the SmPC.

For inclusion, studies were independently selected by three review authors (AM, FO, and EDG) throughout screening titles, abstracts, and keywords. Given the rarity of the disease and lack of robust evidence-based data, case–control studies, case reports, and case series were also considered. Systematic review, basic science studies, meta-analysis, and studies without full text were excluded. Only studies in the English language were included. All included studies were also screened by full articles, and all studies' references were checked for potential inclusion. Disagreements about the selected studies were resolved through discussion between review authors and the senior author (RC), who was an expert in this field.

### Data extraction

The following data were extracted by four authors (AM, EDG, FO, and LP) separately: (1) general information of any article (authors, study design), (2) patients' demographic data, (3) off-label indications for CAN, (4) treatment data (drug posology), (5) treatment response, and (6) adverse events.

All data are included in a predefined data collection form (Table 1).

**Table 1:** Data regarding off-label use of canakinumab in pediatric patients with the immune-mediated disorder from the individual studies included.

**References**	**Study type**	**Sample size**	**Gender (male/female)**	**Disease**	**Dosage**	**Response**	**Adverse events**
Vitale et al. (8)	Case report	1	1/0	Behçet disease	4 mg/kg/4 weeks	Remission	NO
Simonini et al. (9)	Case report	1	1/0	Blau syndrome	2 mg/kg/4 weeks	Remission	NO
PaÇ Kisaarslan et al. (10)	Case report	1	0/1	Blau syndrome	NA	Remission	NO
Bhuyan et al. (11)	Case report	1	1/0	DIRA	4 mg/kg/4 weeks	Ineffective	NO
Kuemmerle-Deschner et al. (12)	Case report	1	0/1	DIRA	150 mg/6 weeks	Remission	NO
Kutukculer et al. (13)	Case report	1	0/1	DIRA	2 mg/kg/4 weeks	Ineffective	NO
Simonini et al. (14)	Case report	1	1/0	Fibrodysplasia ossificans progresiva	300 mg/4 weeks	NA	NO
Barut et al. (15)	Case report	2	1/1	Hyperferritinemic syndromes	4 mg/kg/4 weeks	Remission	NO
Kisla Ekinci et al. (16)	Case report	2	2/0	Majeed syndrome	4 mg/Kg/4 weeks	Remission	NO
Herlin et al. (17)	Case report	1	0/1	Majeed syndrome	2 mg/kg/8 weeks	Remission	NA
Vitale et al. (3)	Retrospective cohort	42	NA	Miscellaneous	2-4 mg/kg/4–8 weeks	NA	NA
Cavalli et al. (18)	Case report	1	0/1	PFAPA	2mg/kg/8 weeks	Ineffective	NO
Mendonça et al. (19)	Case report	1	1/0	PAID	150 mg/4 weeks	Remission	NO
Brambilla et al. (20)	Case series	2	1/1	Uveitis	2 mg/kg/4 weeks	Remission	NO
Sota et al. (5)	Retrospective cohort	111/475	NA	Miscellaneous	2 mg/kg/8 weeks-5 mg/kg/4 weeks	Remission	Mild AE and rare serious AE
Ugurlu et al. (21)	Observational study	NA	NA	Behçet disease	150 mg/6 weeks	NA	NA
Haviv et al. (22)	Case series	2	0/2	CRMO/Majeed syndrome	NA	Remission/ Ineffective	NA
Lopalco et al. (23)	Case report	1	1/0	Recurrent pericarditis	5 mg/kg/ 4 weeks	remission	NO
Kisla Ekinci RM et al. (24)	Case report	1	0/1	Castleman disease	3 mg/kg/ 2 weeks	Ineffective	NA
Kisla Ekinci et al. (16)	Case Report	1	0/1	DADA2	150 mg/ 4 weeks	Remission	NA
Della Casa et al. (25)	Case report	1	1/0	Unclassified autoinflammatory syndromes	4 mg/kg/4 weeks	Ineffective	NA
Pagnini et al. (26)	Case report	1	0/1	Behçet disease	150 mg/8 weeks	Remission	NO
Epçaçan et al. (27)	Case series	2	0/2	Recurrent pericarditis	4 mg/kg/4 weeks-2, 5 mg/kg/single dose	Ineffective	NA
Rees et al. (28)	Clinical trial	49	28/21	Sickle cell anemia	300 mg/4 weeks	Partial remission	NO

AE, adverse events; BS, Behçet syndrome; CRMO, chronic recurrent multifocal osteomyelitis; DADA2, deficiency of adenosine deaminase 2; DIRA, deficiency of interleukin-1 receptor antagonist; NA, not assessed; PAID, PSTPIP1-associated inflammatory diseases; PFAPA, periodic fever, aphthous stomatitis, and pharyngitis cervical adenitis.

## Use of canakinumab in specific diseases

### Monogenic autoinflammatory diseases

The recognition of monogenic disorders with seemingly unprovoked inflammation without the high-titer autoantibodies or antigen-specific T cells usually detected in classic autoimmune diseases gave birth to the concept of autoinflammation (29–31).

The success of IL-1 blockade in cryopyrin-associated periodic syndrome, coupled with the good safety profile of IL-1-inhibiting agents, led to wider use of these agents in a range of monogenic autoinflammatory conditions and also in a number of genetically undifferentiated fever syndromes (32).

Regarding off-label use of CAN in monogenic AIDs, only case reports and case series are available in patients with Majeed syndrome, pediatric granulomatous arthritis (PGA), deficiency of IL-1 receptor antagonist (DIRA), and pyogenic sterile arthritis, pyoderma gangrenosum, acne syndrome (PAPA), deficiency of adenosine deaminase 2 (DADA2), and fibrodysplasia ossificans progressiva (FOP) (9, 11, 12, 14, 16, 17, 22).

Majeed syndrome is an autosomal-recessive disorder characterized by the triad of chronic recurrent multifocal osteomyelitis, congenital dyserythropoietic anemia, and neutrophilic dermatosis that is caused by mutations in *LPIN2*. Herlin et al. first reported the use of CAN (4 mg/kg/4 weeks) in two brothers with Majeed syndrome. Both siblings presented with rapid improvement of symptoms and nearly complete resolution of the bone lesions on MRI after 3 months (17).

A significant clinical and laboratory improvement was also detected in two other patients with Majeed syndrome. The first patient represented the first report of an American patient found to be compound heterozygous and received CAN at 2 mg/kg every 8 weeks (11). The second patient was treated with monthly CAN after the development of dyserythropoietic anemia, resulting in clinical improvement within 3 months of treatment (33).

The familiar and sporadic forms of PGA, respectively called Blau syndrome and early-onset sarcoidosis, are caused by autosomal-dominant gain-of-function mutations in the *NOD2* gene. Their clinical picture is characterized by the triad of granulomatous polyarthritis, panuveitis, and granulomatous exanthema.

Given disease rarity, specific therapeutic guidelines are currently lacking. Tumor necrosis factor (TNF) inhibitors, mainly monoclonal antibodies, are the most commonly used agents among biologics. In contrast, IL-1 blockade has been employed with varying degrees of success, usually as a second option after the failure of TNF-α inhibitors (34). Evidence of CAN in PGA remains anecdotic. Simonini et al. reported a 16-year-old boy who was diagnosed with Blau syndrome at the age of 4 years, treated with systemic and topical steroids, as well as several disease-modifying agents, including methotrexate, infliximab, adalimumab, mycophenolate mofetil, and abatacept, without significant improvement. Due to disease flares, he underwent a trial of CAN (2 mg/kg/month). During the next 6 months, no ocular flares occurred and no steroid pulse therapy was necessary (14). In a cross-sectional multicenter study, CAN was employed in association with methotrexate and glucocorticoids (GCs) in one patient with Blau syndrome, yielding control of the articular involvement (35). Other two patients were treated with CAN in a small Turkish case series of six patients with PGA, one achieving remission while another was found unresponsive (10).

Papatesta et al. reported on a 7-year-old Caucasian girl with Blau syndrome first misclassified as juvenile idiopathic arthritis, which was unresponsive to etanercept and tocilizumab. CAN therapy associated with GCs led to a prompt clinical improvement and normalization of laboratory markers. After 12 months of CAN monotherapy, she was in complete clinical and laboratory remission. The patient experienced a substantial improvement in quality of life while no serious adverse event occurred (36).

Deficiency of IL-1 receptor antagonist (IL-1Ra) (DIRA) represents a very rare AID where mutations in the *ILR1N* gene result in a severe systemic inflammation manifesting as perinatal-onset pustular dermatitis, multifocal nonbacterial osteomyelitis, periostitis, arthritis, and elevated acute-phase reactants. CAN use in patients with DIRA has displayed different outcomes in terms of efficacy. Ulusoy et al. described the first patient with DIRA with an excellent response to CAN. This was a case of late-onset DIRA syndrome arising in a 12-year-old girl treated with 150 mg once every 6 weeks with progressive resolution of clinical signs in 12 months and no adverse events reported (9). In contrast, three case reports have evidenced a partial-to-poor response to CAN, resulting in disease flare (12, 13, 19). Interestingly, the therapeutic switch to anakinra resulted in complete control of disease activity and dramatic regression of bone inflammation (12, 19).

Mutations of the *PSTPIP1* gene were associated with a large group of inflammatory disorders collected under the term PSTPIP1-associated inflammatory diseases (PAID). Borgia et al. reported a case of a patient carrying the *PSTPIP1* p.E250K mutation who developed a late-onset kidney involvement despite a long treatment with CAN. The authors pointed out that specific organ impairment may be independent of anti-cytokine treatment, highlighting the need for a periodic diagnostic workup (37).

DADA2 is a rare AID due to the mutation of the cat eye chromosome region 1 (*CECR1*) gene encoding for adenosine deaminase 2, clinically characterized by early-onset strokes, livedo reticularis, and periodic fever. Usually, treatments of the disease include systemic steroids, immunosuppressive drugs such as azathioprine, methotrexate, calcineurin inhibitors, and cyclophosphamide, and biological drugs such as anti-TNF agents (38).

Kisla Ekinci et al. described a case of a 13-year-old female patient presenting with generalized edema, hepatosplenomegaly, left knee arthritis associated with renal amyloidosis, and low serum immunoglobulin G and immunoglobulin M levels. DADA2 diagnosis was suspected and then genetically confirmed, and she started treatment with colchicine and methylprednisolone without any benefit in terms of improvement in proteinuria. After 2 months, she started therapy with CAN with a successful outcome of clinical and laboratory parameters (16).

FOP is a rare genetic disorder caused by mutations in the *ACVR1/ALK2* gene (39). Haviv et al. described a case of a 13-year-old boy with FOP who presented with asymmetrical shoulder position, limited rotation of the neck, spine, and left hip, and abnormally short and wide halluces. The patient had generalized osteopenia, several chondrocalcinosis and non-homogenously edematous muscles, thickening of the submental fascia, and infiltration of subcutaneous fat on MRI. First, he was unsuccessfully treated with high-dose corticosteroids, pamidronate infusions, celecoxib, and montelukast. Therefore, he first started anakinra with a notable improvement in the disease, and then was successfully treated with CAN due to high levels of IL-1β found in the patient's plasma samples, collected during a paroxysm (22).

### Polygenic autoinflammatory diseases

Polygenic AIDs are designated as a category of complex multifactorial diseases of unknown etiology characterized by the overexpression of inflammasome-associated genes that lead to a dysregulated innate immune response (40). BS presents with a protean clinical picture and complex pathogenesis. IL-1 was found to be a crucial proinflammatory cytokine in the complex BS pathogenesis and might represent a promising target among novel therapeutic approaches (41). Increased levels of IL-1β were detected among patients with active disease, and those levels correlated with the imbalance of the oxidant/antioxidant system (42).

Evidence of CAN effectiveness in BS derives from data collected on adult patients. Emmi et al. evaluated the efficacy, safety profile, and timing of the response to the therapy in 30 patients with BS receiving treatment with anti-IL-1 agents (anakinra and CAN). Data confirmed that the use of anti-IL-1 drugs is efficacious and presents a good safety profile (43). Regarding specific organ involvement, uveitis has been found to predict a good and sustained response to IL-1 inhibitors (44), also in patients with long-standing disease and refractory to other immunosuppressant agents (3). Vitale et al. described a case series of adults with refractory uveitis in BS responders or maintaining remission on CAN in monotherapy (150 mg subcutaneous monotherapy every 6 weeks), after steroid and immunosuppressive drugs failure or anakinra discontinuation due to side effects (8).

However, in patients with juvenile-onset of BS and pediatric uveitis related to BS, reported data on treatment with CAN are anecdotal. Pagnini et al. described a case of a severe patient with BS who was refractory to treatment with traditional immunosuppressive drugs and different biological agents but was successfully treated with CAN. In detail, the patient presented with recurrent fever, oral and genital ulcerations, and skin lesions, headache associated with constipation, abdominal pain, and arthralgia. First, he was treated with colchicine, then thalidomide plus prednisone. Due to the persistence of symptoms, the treatments, including mycophenolate mofetil, adalimumab, and anakinra, were trialed without clinical benefit. Finally, the patient received CAN (4 mg/kg every 4 weeks) with complete clinical and laboratory remission (26).

Ugurlu et al. reported a severe case of bilateral panuveitis and retinal vasculitis in a patient with pediatric BS refractory to multiple treatments such as azathioprine, cyclosporine A, infliximab, adalimumab, and anakinra. The patient was then treated with a single dose of CAN 150 mg and was reported to be attack-free in the following 8 weeks (21).

Finally, with regard to the safety profile of CAN in patients with BS, Cantarini et al. conducted a multicenter observational study to examine the overall safety profile of biological treatments. In detail, they enrolled 85 patients with BS who were treated with TNF-α inhibitors (67/85) or anti-IL-1 (18/85) as the first, second, or third biological line of treatment according to the best standards of care. Data confirmed that both TNF-α inhibitors and anti-IL-1 agents have a good efficacy and safety profile (45).

Idiopathic recurrent pericarditis (IRP) represents a debilitating complication of acute pericarditis and displays a strong autoinflammatory component from a pathogenetic standpoint (23).

Its management relies on nonsteroidal anti-inflammatory drugs that remain the mainstay of treatment. Colchicine is recommended to improve remission rates and prevent recurrences. Patients failing standard therapy or those with GC dependence may benefit from IL-1 inhibition with anakinra, which has yielded promising results also in one randomized clinical trial (23).

The efficacy of CAN in IRP on a large scale has yet to be evaluated. To date, only a few case reports or case series disclosing controversial results are available in the literature.

A 6.5-year-old child with GC-dependent IRP showed complete response to CAN after anaphylaxis occurring during treatment with anakinra (27). Signa et al. describe two cases of failure of the treatment with an anti-IL-1β monoclonal antibody in steroid-dependent IRP, successfully managed with anakinra (46).

A preliminary multicenter Italian experience on a pediatric cohort has suggested that the ability of CAN to control recurrences does not seem as high as that of anakinra. A total of five patients were treated with CAN: one as the first anti-IL1 drug and four were switched from anakinra. Of the five patients, two had complete control of the diseases, two discontinued the treatment because of inefficacy, and one required a low dose of GCs to control the disease (47). Altogether, these data suggest a pivotal role of IL-1α in the pathogenesis of IRP (47). Indeed, IL-1α and IL-1β maintain nonredundant inflammatory functions and display some differences in terms of biological properties (18).

PFAPA syndrome is the most common cause of periodic fever in childhood. Soylu et al. presented a report of a child presenting with recurrent, self-limited febrile attacks at 1 year of age who was diagnosed with familial Mediterranean fever being heterozygous for *M694V* mutation. Her clinical findings were only controlled by the addition of CAN (2 mg/kg/8 week) on top of colchicine treatment. However, she developed typical PFAPA attacks during this treatment at 3 years of age, and IL-1β blockade with CAN did not prevent the occurrence of PFAPA attacks (48).

Finally, there is a group of diseases, named unclassified AIDs, that do not fit in any of the clinically defined systemic autoinflammatory conditions and do not have any known pathogenetic mutations in genes (25). For this reason, the treatment of these diseases is based on expert opinion and similarities with other diseases (49).

Tucker et al. presented a case of a 4-year-old boy with recurrent rash and fever since the age of 18 months, associated with lethargy, arthralgia, myalgia, headache, cough, vomiting, and loose stool. At the age of 6 years, he developed an episode of macrophage activation syndrome (MAS). Hence, an extensive analysis of genetic investigation was performed, and numerous variants of unknown significance were identified in genes (*MEFV* and *NLRP12*) known to be associated with AIDs. First, he was treated with colchicine, and then with steroids and anakinra, with minimal clinical improvement. Therefore, CAN therapy was started with rapid and complete resolution of fever and, partially, of the signs and symptoms associated (50).

### Hyperferritinemic syndromes

MAS is a variant of reactive hemophagocytic lymphohistiocytosis associated with a high mortality rate and characterized by cytopenia, organ dysfunction, and coagulopathy associated with inappropriate activation of macrophages (51). Although systemic GCs and cyclosporine A remain the therapeutic cornerstones, MAS is often unresponsive and other treatment options should be exploited. Contrary to the widespread agreement that anakinra, particularly at high doses, might be effective in patients with MAS, the role of CAN in this potentially fatal condition is less clear. Additionally, the occurrence of MAS in systemic-onset juvenile idiopathic arthritis (sJIA) treated with CAN in randomized clinical trials has raised some concerns. Nevertheless, CAN does not appear to increase the overall risk of MAS or affect its clinical features (52, 53). In support of the latter, long-term surveillance data from the German BIKER registry on biological therapies in sJIA did not detect significant differences between CAN, tocilizumab, anakinra, and etanercept in terms of MAS occurrence (54).

In addition, case series and single case reports have reported encouraging results on patients diagnosed with MAS and treated with CAN (15, 55–57).

Barut et al. studied 10 patients with MAS followed up for 1 year with four receiving CAN and five anakinra. Clinical improvement was observed in all patients except one, who showed recurrent MAS attacks (15). An excellent response to CAN in association with sildenafil was obtained in a patient with sJIA complicated with MAS and pulmonary hypertension (57). Papa et al. described two pediatric patients with hyperferritinemic syndromes refractory or intolerant to conventional therapies. The administration of CAN (4 mg/kg monthly in one patient and 5 mg/kg in the other patient) led to rapid recovery of all clinical and laboratory features of MAS (56). CAN may indeed represent an alternative therapeutic option for pediatric patients with MAS who are refractory or intolerant to conventional therapies (56).

### Other diseases

#### Kawasaki disease

Kawasaki disease (KD) is an acute inflammatory vasculitis associated with a significantly high risk of coronary artery abnormalities. Hashimoto et al. investigated the effects of an anti-IL-1β antibody using a *Candida albicans* water-soluble fraction (CAWS)-induced mouse model of KD evidencing significant attenuation of CAWS-induced vasculitis (58). No human data are available so far.

#### Uveitis

Noninfectious uveitis represents a rare condition in the pediatric population, mostly associated with JIA (59). Compared to adult uveitis, the pediatric form is more commonly asymptomatic while having the tendency to become chronic; indeed, up to 90% of pediatric uveitis cases present as chronic anterior uveitis and are associated with a significant rate and spectrum of sight-threatening complications (20, 60–63).

Treatment of noninfectious uveitis is currently based on GCs that are mainly used in the acute phase during a disease flare or as a bridge therapy to GC-sparing agents. The advent of biotechnological agents has revolutionized the management of noninfectious uveitis (64). In particular, a robust experience has accrued over time with TNF-α inhibitors both in a randomized clinical trial and in a real-life setting (60, 61, 65–68). Other mechanisms of action, including IL-6 inhibition, as well as CD20 and costimulatory blockade, have been employed with promising results (62). Data regarding CAN efficacy in noninfectious pediatric uveitis in an off-label context are limited to small case series and single case reports.

Brambilla et al. described two cases of severe pediatric sight-threatening uveitis, one JIA-related recurrent uveitis and the other classified as idiopathic uveitis, both refractory to previous biological therapies. Patients achieved remission after treatment with CAN (2 mg/kg monthly). Treatment with CAN was also able to improve visual acuity while allowing a GC-sparing effect (20). As mentioned in the “Monogenic autoinflammatory diseases” section, Simonini et al. described a patient with Blau syndrome and severe ocular involvement (granulomatous retinal lesions, anterior chamber inflammation, and complications like macular edema leading to retinal detachment). Treatment with CAN was well tolerated and yielded a rapid quiescence of uveitis that has been unresponsive to previous treatment, including different biological agents (14). In such scenarios, CAN may constitute a feasible alternative for pediatric uveitis refractory to other immunosuppressive treatments.

#### Castleman disease

Castleman disease (CD) is a group of lymphoproliferative disorders uncommon in pediatric age and classified according to the number of regions of enlarged lymph nodes involved.

Clinically, the CD is grouped into two categories as unicentric CD (UCD) and multicentric CD (MCD) and, according to the histology, into three subtypes such as hyaline vascular variant, plasma cell variant, and mixed variant (69). Data regarding CD management are scarce, given the rarity of the disease, and there are no evidence-based recommendations on the treatment of pediatric CD.

Patients with UCD are usually surgically treated with complete resection of the pathological lymph node. Treatment of MCD cases is based on adult experiences and consists of anti-IL-6 agent use or alternative options with GCs, rituximab, or conventional cytotoxic chemotherapy (70).

Kisla et al. reported a case of a 6-year-old girl with recurrent fever associated with abdominal pain and persistently elevated inflammatory markers, previously diagnosed as unclassified AIDs syndrome resistant to colchicine, then treated with CAN and steroids. Unfortunately, symptoms and inflammatory markers did not improve with treatment; therefore, she underwent a positron emission tomography scan, which showed a circumscribed intrarenal, retroperitoneal mass, and millimetric axillary and cervical lymph nodes, confirming a UCD diagnosis. After an excisional biopsy by laparotomy, the inflammatory markers were normalized and remained normal after 2 months without any relapses (24).

#### Sickle cell anemia

Sickle cell anemia (SCA) is a rare and severe inherited disorder due to a point mutation in the β globin gene changing a glutamic acid to valine. It results in sickle hemoglobin with a tendency to polymerize when deoxygenated. Clinically, SCA signs include anemia, chronic pain, and fatigue with episodic disease flares of acute vaso-occlusive events. Most recent evidence supports the hypothesis that inflammasome is constantly active in patients with SCA, causing exaggerated, pro-inflammatory responses after a disease flare (71, 72).

Based on these hypotheses, Rees et al. suggested that the use of CAN in patients with SCA could reduce inflammation and clinical disease activity. The authors conducted a randomized, double-blind, multicenter phase 2a study enrolling patients with SCA between 8 and 20 years of age. Patients were randomized 1:1 to receive 6-monthly treatments with 300 mg subcutaneous CAN or placebo. Although the primary objective (reduction of pain) was not met, patients in the CAN arm showed a reduction in inflammation markers and the occurrence of SCA-related flares, and therefore, the reduction of numbers and duration of hospitalizations, the trend for a decrease in pain, fatigue, and absences from school or work.

Regarding safety, CAN was well tolerated with no drug-related adverse events (28).

## Conclusion

The efficacy and safety of CAN employed in an off-label fashion have been reported in pediatric patients affected by several immune-mediated disorders unresponsive to standards of care, suggesting the important role of IL-1β in their pathogenesis. Broadly speaking, a significant improvement in most diseases has been detected, resulting in good clinical response. Promising results have been obtained in monogenic and polygenic AIDs, hyperferritinemic syndromes, and intraocular inflammation. Moreover, recent studies suggest the selective blockade of IL-1β and the involvement of inflammasome also in some rare diseases, inspiring a fascinating perspective. This is of paramount importance in pediatric patients, where a considerable proportion of treatments are prescribed off-label.

Nevertheless, caution is needed when interpreting the data collected since a considerable amount of evidence comes from case series and single-patient reports. Powered studies properly designed are warranted to draw firm conclusions regarding CAN effectiveness, its appropriate posological regimen for each disease, as well as treatment duration in these diseases.

## Data availability statement

The original contributions presented in the study are included in the article/supplementary material, further inquiries can be directed to the corresponding author.

## Author contributions

ED, JS, and AM drafted the manuscript and gave final approval for the version to be published. LP, FO, ED, and AM collected and interpreted the data and revised the manuscript. RC and LC supervised and revised the manuscript critically for important intellectual content. All authors approved the final manuscript as submitted.

## Conflict of interest

The authors declare that the research was conducted in the absence of any commercial or financial relationships that could be construed as a potential conflict of interest.

## Publisher's note

All claims expressed in this article are solely those of the authors and do not necessarily represent those of their affiliated organizations, or those of the publisher, the editors and the reviewers. Any product that may be evaluated in this article, or claim that may be made by its manufacturer, is not guaranteed or endorsed by the publisher.
